# Effect of prior receipt of antibiotics on the pathogen distribution: a retrospective observational cohort study on 27,792 patients

**DOI:** 10.1186/s12879-019-4724-6

**Published:** 2020-01-06

**Authors:** Leiqing Li, Lingcheng Xu, Rongsheng Zhu, Jiaojiao Song, Xuanding Wang

**Affiliations:** 1 Department of Critical Care Medicine, the Second Affiliated Hospital of Zhejiang University School of Medicine, Hangzhou, 310009 China; 2 Department of Infection Prevention and Control, the Second Affiliated Hospital of Zhejiang University School of Medicine, Hangzhou, 310009 China

**Keywords:** Clinical specimen, Microorganism distribution, Antimicrobial stewardship

## Abstract

**Background:**

There have been no systematic studies of microbiological differences before and after antibiotics treatment. The aim of this study was to evaluate the effect of prior receipt of antibiotics on the microorganism distribution.

**Methods:**

A retrospective, observational cohort study was conducted in a 3200-bed tertiary, referral, teaching hospital in eastern China. During a 2-year period, all hospitalized patients treated with antimicrobial agents were enrolled in this study. Among 48,692 patients evaluated, the 27,792 (57.1%) who were sampled within 2 days before or after administration of the first dose of antimicrobial agents were included. Distribution of clinical specimens and the microorganism were compared between before and after antibiotic drug treatment groups.

**Results:**

Compared to specimens taken after antibiotics exposure, specimens taken before antibiotics exposure had a higher proportion of blood and urine specimens and a higher culture positive rate (all *P* < 0.001). Higher percentages of *Staphylococcus aureus* (9.9% vs. 8.5%, *P* = 0.041), non-fermenting bacteria (27.7% vs. 19.9%, *P* < 0.001), and fungi (8.4% vs. 4.0%, *P* < 0.001) were isolated from the group after antibiotics exposure, while the percentages of *Streptococcus spp.* (4.8% vs. 2.7%, *P* < 0.001), *Haemophilus influenzae* (2.3% vs. 0.8%, *P* < 0.001), and *Moraxella catarrhalis* (0.7% vs. 0.1%, *P* < 0.001) were higher in the group before antibiotics exposure. Further analysis found significant differences of microbes derived from respiratory secretions, blood or urine samples. We found, after antibiotics exposure, the separation rate of non-fermenting bacteria was significantly increased (all *P* < 0.05), and the separation rate of *Candida spp*. was higher, with statistical significance in airway secretion and urine samples (both *P* < 0.05), but the separation rate of *Staphylococcus aureus* among the three groups was not affected by antibiotics. In addition, the isolation rate of *Streptococcus spp.* in blood and urine samples decreased significantly (both *P* < 0.05) after antibiotics exposure. Interestingly, no statistical difference was found for microbes isolated from body fluid specimens between the two groups.

**Conclusions:**

The outcome revealed that antibiotic-insensitive organisms such as non-fermentative bacteria and fungi were more frequently isolated after antibiotics exposure. However, this trend might be specimen dependent and was not obvious in body fluid specimens.

## Background

Antimicrobial resistance has increased in recent decades. Antimicrobial stewardship is important for addressing the problem of resistant pathogens, and obtaining specimens prior to the administration of antimicrobials is a key part of this program. Sterilization of cultures may occur shortly after the use of antibiotics [1, 2], which may lead to negative culture results as described in some research [3–9]. Moreover, studies conducted by Montravers et al. [10] and Harbarth et al. [11] showed that starting antibiotic therapy before sample collection may result in detecting less-sensitive microorganisms. However, the former only included 76 consecutive patients with ventilator-associated pneumonia [10], and the latter [11] mainly evaluated gram-negative pathogens. In addition, one study assessed the impact of prebiopsy antibiotics on pathogen recovery in hematogenous vertebral osteomyelitis patients and revealed that antibiotic exposure before biopsy did not negatively impact pathogen recovery [12]. Therefore, a systematic study of the microbiological differences isolated from samples taken before and after the initiation of antibiotic therapy, which to the best of our knowledge is lacking in the literature, is necessary.

Here, we developed a computerized antimicrobial decision-support system (aCDSS) embedded in our hospital’s electronic medical record system (EMRS) that integrated into the clinical workflow required doctors obtain microbiological specimens before antibiotic therapy starting in 2015. With the assistance of aCDSS, we were able to classify all clinical specimens according to collection time and record the microorganisms that were present, thus analyzing the effects of antimicrobial agents on the distribution of organisms. The overall aim of this study was to assess the potential association between antimicrobial agent therapy and the distribution of microbes isolated from clinical specimens.

## Methods

### Study design and setting

This study was performed at the second affiliated hospital of Zhejiang University School of Medicine, a 3200-bed tertiary referral and teaching hospital in China. The protocol was approved by human research ethics committee of the second affiliated hospital of Zhejiang University School of Medicine (2018–025). Due to the retrospective observational nature of the study, the Institutional Review Board waived the need for informed consent.

All hospitalized patients treated with antibiotics discharged from 1 January 2015 to 31 December 2016 in our hospital were enrolled. Patients who had clinical specimens collected within 2 days before or after antibiotic therapy were included, while those who did not have clinical specimens collected during this period were excluded. Clinical specimens of infection sources were obtained as clinically indicated, but only the first sample (infection episode) during this period (within 4 days as described above) per patient was included in the analysis.

Microbial diagnostic processing included specimen collection, smear stain, screening, primary culture media selection. The main media were including blood agar, chocolate agar, China blue (prussian blue and Rhodizonic acid sodium) agar, Salmonella Shigella agar(for fecal specimens), Sabouraud agar and CHROM agar candida (for fungi), etc. Blood agar, chocolate agar, etc. for fastidious bacteria were placed into 35 °C and 5% CO_2_ incubator, other media for bacteria were placed into 35 °C atmospheric incubator, fungal media were placed into 28 °C, 80–90% RHs atmospheric incubator. Incubation period were 18–24 h for most specimens to bacteria, and 24–72 h for fungi, delayed in special situations.

With the assistance of aCDSS, we recorded the accurate time of the first dose of therapeutic antibiotics for each infected (or suspected to be infected) patient and the time that the specimen was taken, as well as the corresponding microorganism results. A polymicrobial result was defined as more than one pathogen cultured from the same specimen. We also took executive infection control measures during this study period in our hospital. All of the patients were treated by the attending physician.

### aCDSS

The aCDSS embedded in our hospital’s EMRS was developed specifically to target antibiotics, including prophylaxis and therapeutic use, and was linked to the hospital laboratory information system in real-time to provide physicians with the latest patient information. When prescribing antibacterial drugs, the system automatically decides whether the purpose is for “therapeutic” or “prophylactic” use according to the repository set up by our research group. For therapeutic use, the aCDSS integrated into the clinical workflow requires doctors to obtain microbiological specimens before starting antibiotic therapy. In addition, nurses need to use a personal digital assistant to scan a two-dimensional code of samples before using antimicrobial drugs. Therefore, aCDSS can accurately record the time of antibiotic use as well as the time of sample taken.

### Data collection

Cases were assigned randomly to 1 of 3 trained extractors, who extracted demographic data. Subjects were assigned to two groups according to the time sequence of sampling and the first dose of antibiotics: the SBA group (specimen taken before antibiotic therapy) and SAA group (specimen taken after antibiotic therapy). Department distribution of patients was divided into 4 groups: Intensive Care Unit (ICU); surgical departments, including department of General Surgery, Thoracic Surgery, Cardiovascular Surgery, Urology Surgery, Vascular Surgery, Neurosurgery, Orthopedics, Oncology Surgery, Gynecology, Obstetrics, Ophthalmology, Otolaryngology, Plastic Surgery, Oral Surgery and Burns; internal departments, including department of Gastroenterology, Cardiology, Respiratory Medicine, Endocrinology, Hematology, Nephrology, Infectious Diseases, Medical Oncology, Radiation Oncology, Neurology, Pediatrics, Psychiatry, Rheumatology and Dermatology; others, including department of Emergency, General practice, Rehabilitation and Traditional Chinese Medicine.

### Statistical analysis

All statistical analyses were conducted using MS Excel 2016. Data are expressed as means ± standard deviation (SD) or medians (interquartile range, IQR) for continuous variables, and as frequencies (percent) for categorical variables. Groups were compared using Student’s *t* test or analysis of variance (ANOVA) for continuous variables and the chi-square (χ^2^) test or Fisher’s exact test for categorical data as appropriate. All statistical tests were 2-tailed, and *P* < 0.05 indicated statistical significance.

## Results

During the study period, a total of 48,692 patients were treated with various antibiotics, with samples taken from 27,792 (57.1%) patients within 2 days before or after starting antibiotic treatment. Among them, 19,868 (71.5%) of patient samples were taken before antibiotic therapy (SBA group), and 7924 (28.5%) of patient samples were taken after antibiotic therapy (SAA group).

### Patient characteristics and culture positive rate

The demographic characteristics of the included patients are shown in Table 1. Patients in the SAA group were older than those in the SBA group (*P* < 0.001). And both groups had a male predominance (62.8% in the SBA group and 62.3% in the SAA group; *P* = 0.443). Compared with the SAA group, the SBA group included more patients from ICU and internal departments(both *P* < 0.001), with less subjects from surgery departments and other departments (both *P* < 0.001), indicating patients from the latter departments were more likely to be exposed to antibiotics before clinical specimens collected.

**Table 1:** Characteristics, clinical specimen distribution, and culture positive rate of SBA and SAA groups

	SBA group *N* = 19,868	SAA group *N* = 7924	*P* value
Demographic			
Age in yr, medians (IQR)	60 (47–70)	61(49–72)	< 0.001
male (%)	12,477 (62.8%)	4938 (62.3%)	0.443
Department distribution			
Intensive Care Unit	1582 (8.0%)	499 (6.3%)	< 0.001
Surgery departments	8760 (44.1%)	3873 (48.9%)	< 0.001
Internal departments	8849 (44.5%)	3114 (39.3%)	< 0.001
Others	677 (3.4%)	428 (5.5%)	< 0.001
Distribution of Clinical specimens			
Respiratory specimens ^a^	7351 (37.0%)	3162 (39.9%)	< 0.001
Blood	4229 (21.3%)	1196 (15.1%)	< 0.001
Body fluid specimens ^b^	1495 (7.5%)	1131 (14.3%)	< 0.001
Urine	4311 (21.7%)	947 (12.0%)	< 0.001
Stool	403 (2.0%)	297 (3.7%)	< 0.001
Other specimens ^c^	2079 (10.5%)	1191 (15.0%)	< 0.001
Culture positive rate			
Culture positive, n (%)	5650 (28.4%)	2014 (25.4%)	< 0.001
Polymicrobial result, n (%)^d^	864 (15.3%)	320 (15.9%)	0.525

Abbreviations: *SBA* specimen taken before antibiotic therapy, *SAA* specimen taken after antibiotic therapy

^a^ including sputum, throat swab, tracheal aspirate, protected bronchial brush, and bronchoalveolar lavage

^b^ including cerebrospinal fluid, pleural fluid, ascites, bile, puncture fluid, and pericardial effusion

^c^ including intravascular catheter tip, various secretions, peritoneal dialysate, pus, drainage fluid, biopsy tissue and other specimens

^d^ Polymicrobial result was defined as more than one pathogen cultured from the same specimen

The microbiological results suggested that the culture positive rate in the SBA group was higher than that of the SAA group (28.4% vs. 25.4%; *P* < 0.001), but there was no significant difference in the proportion of polymicrobial results between the two groups (15.3% in the SBA group vs. 15.9% in the SAA group; *P* = 0.525).

### Antimicrobial used in SAA groups

**Fig. 1:**
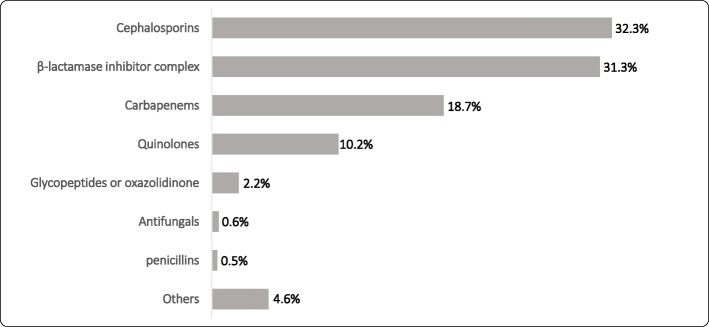
Antimicrobial classification prescribed before sampling in SAA group. Abbreviations: SAA, specimen taken after antibiotic therapy

We classified antimicrobial drugs (either intravenously or orally) prescribed before sampling in SAA group, as shown in Fig. 1. The common antibiotics used in SAA group were Cephalosporins (32.3%), β-lactamase inhibitor complex (31.3%), Carbapenems (18.7%), and Quinolones (10.2%).

### Clinical specimen distribution

There was a significant difference in clinical specimen distribution between the two groups as shown in Table 1. The proportion of blood and urine specimens taken from the SBA group was higher than that of the SAA group (21.3% vs. 15.1 and 21.7% vs. 12.0%, respectively, both *P* < 0.001), while the proportion of respiratory secretions (including sputum, throat swab, tracheal aspirate, protected bronchial brush, and bronchoalveolar lavage), body fluid specimens (including cerebrospinal fluid, pleural fluid, ascites, bile, puncture fluid, and pericardial effusion), stool, and other specimens (including intravascular catheter tip, various secretions, peritoneal dialysate, pus, drainage fluid, biopsy tissue and other specimens) sampled from the SBA group were lower than those of the SAA group with significance (37.0% vs. 39.9%, 7.5% vs. 14.3%, 2.0% vs. 3.7%, 10.5% vs. 15.0%, respectively, all *P* < 0.001).

### Microbial distribution isolated from specimens

Final culture results were made available by aCDSS, which yielded a total of 8850 isolates, with 6516 strains isolated from the SBA group and 2334 from the SAA group. Differences in microbial distribution between the two groups are shown in Table 2.

**Table 2:** Distribution of microorganisms isolated from specimens taken from SBA and SAA groups

Organisms	No. of Isolates in SBA Group (*N* = 6516), n (%)	No. of Isolates in SAA Group (*N* = 2334), n (%)	*P* value
Bacteria	6256 (96.0%)	2138 (91.6%)	< 0.001
Gram-positive organism	1773 (27.2%)	577 (24.7%)	0.020
*S. aureus*	556 (8.5%)	232 (9.9%)	0.041
CNS	469 (7.2%)	105 (4.5%)	< 0.001
*Streptococcus spp.*	311 (4.8%)	64 (2.7%)	< 0.001
*Enterococcus spp.*	437 (6.7%)	176 (7.5%)	0.173
Gram-negative organism	4268 (65.5%)	1515 (64.9%)	0.607
Enterobacteriaceae	2777 (42.6%)	847 (36.3%)	< 0.001
*E. coli*	1008 (15.5%)	226 (9.7%)	< 0.001
*K. pneumoniae*	1037 (15.9%)	375 (16.1%)	0.863
*Enterobacter spp*.	237 (3.6%)	125 (5.4%)	< 0.001
Other Enterobacteriaceae bacteria	495 (7.6%)	121 (5.2%)	< 0.001
Non-fermenting bacteria	1296 (19.9%)	647 (27.7%)	< 0.001
*Pseudomonas spp.*	569 (8.7%)	236 (10.1%)	0.047
*Acinetobacter spp.*	487 (7.5%)	274 (11.7%)	< 0.001
*Burkholderia spp.*	32 (0.5%)	17 (0.7%)	0.185
*S. maltophilia*	114 (1.7%)	61 (2.6%)	0.01
Other non-fermenting bacteria	94 (1.4%)	59 (2.5%)	< 0.001
*H. influenzae*	150 (2.3%)	19 (0.8%)	< 0.001
*M. catarrhalis*	45 (0.7%)	2 (0.1%)	< 0.001
Other bacteria	215 (3.3%)	46 (2.0%)	0.001
Fungi	260 (4.0%)	196 (8.4%)	< 0.001
*Candida spp.*	233 (3.6%)	185 (7.9%)	< 0.001
*Aspergillus spp.*	20 (0.3%)	10 (0.4%)	0.386
*Cryptococcus spp.*	3 (0.0%)	0 (0.0%)	0.571^*^
Other fungi	4 (0.0%)	1 (0.0%)	0.854^*#*^

Abbreviations: *SBA* specimen taken before antibiotic therapy, *SAA* specimen taken after antibiotic therapy, *S. aureus Staphylococcus aureus,* CNS coagulase-negative Staphylococcus, *E. coli Escherichia coli, K. pneumoniae Klebsiella pneumoniae, S. maltophilia Stenotrophomonas maltophilia, H. influenzae Haemophilus influenzae, M. catarrhalis Moraxella catarrhalis*

*Fisher’s exact test

^*#*^ Corrected chi-square test

The proportion of bacteria in the SBA group was 96.0% (6256 strains), higher than that of the SAA group (91.6%, 2138 strains, *P* < 0.001); while the proportion of fungi isolated from the SAA group was 8.4%, higher than that of the SBA group (4.0%, *P* < 0.001). The ratio of gram-positive bacteria isolated from the SBA group was slightly more than that isolated from the SAA group (27.2% vs. 24.7%, *P* = 0.020), while separation rate of gram-negative bacteria was not statistically different between the two groups (65.5% vs. 64.9%, *P* = 0.607).

### Gram-positive microorganisms

The percentage of *Staphylococcus aureus (S. aureus)* in the SAA group was higher than that in the SBA group (9.9% vs. 8.5% *P* = 0.041). In contrast, the isolation rates of coagulase-negative Staphylococcus (CNS) and *Streptococcus spp.* in the SBA group were higher than those in the SAA group (7.2% vs. 4.5% and 4.8% vs. 2.7% respectively, both *P* < 0.001). No significant difference in *Enterococcus spp.* was observed between the two groups (*P* = 0.173).

### Gram-negative microorganisms

The percentage of Enterobacteriaceae isolated from the SBA group was higher than that from the SAA group (42.6% vs. 36.3%, *P* < 0.001). In contrast, the isolation rate of non-fermenting bacteria in the SBA group was lower than that in the SAA group with significant difference (19.9% vs. 27.7%, *P* < 0.001). In the Enterobacteriaceae, the isolation rate of *Escherichia coli (E. coli)* in the SBA group was higher than that in the SAA group (15.5% vs. 9.7%, *P* < 0.001), while the isolation rates of *Enterobacter spp.* in the SBA group was lower than those in the SAA group (3.6% vs. 5.4%, *P* < 0.001). No significant difference in *Klebsiella pneumoniae* (*K. pneumoniae)* was observed between the two groups (*P* = 0.863).

As for non-fermenting bacteria, the isolation rates of *Pseudomonas spp.*, *Acinetobacter spp.*, and *Stenotrophomonas maltophilia (S. maltophilia)*, were significantly lower in the SBA group than those in the SAA group (8.7% vs. 10.1%, 7.5% vs. 11.7%, and 1.7% vs. 2.6%, respectively, all *P* < 0.05).

Although rarely isolated, the separation rates of *Haemophilus influenzae (H. influenzae)* and *Moraxella catarrhalis (M. catarrhalis)* between the two groups were significantly different (2.3% in the SBA group vs. 0.8% in the SAA group and 0.7% in the SBA group vs. 0.1% in the SAA group, respectively, both *P* < 0.001).

### Fungi

The isolation rate of *Candida spp.* in the SAA group was 7.9%, which was higher than that in the SBA group (3.6%, *P* < 0.001). None of the other species had significant differences between the two groups.

### Subgroup analysis

As a result of inconsistent sample composition between the two groups, which may lead to bias, we conducted subgroup analysis to compare the microorganism distribution according to the specimen types.

### Respiratory secretions

**Fig. 2:**
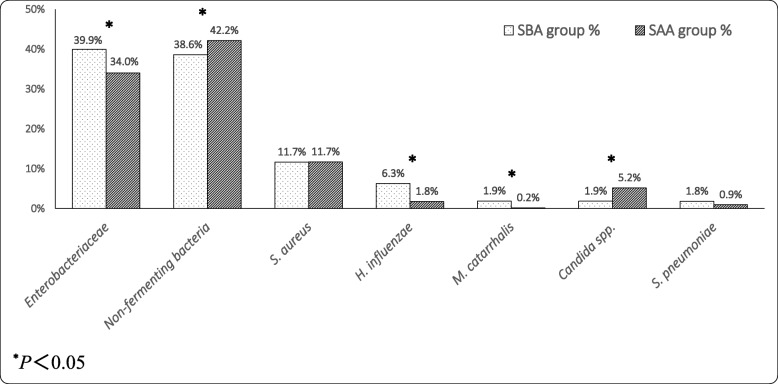
Comparison of microorganisms isolated from respiratory secretions between SBA (top 7) and SAA groups. Abbreviations: SBA, specimen taken before antibiotic therapy; SAA, specimen taken after antibiotic therapy; *S. aureus, Staphylococcus aureus; H. influenzae, Haemophilus influenzae; M. catarrhalis, Moraxella catarrhalis; S. pneumoniae, Streptococcus pneumoniae*

Further analysis of respiratory secretions found that the microbial ratio was significantly different between the two groups. Figure 2 shows the descending order of the top 7 pathogens of the SBA group (dotted columns) corresponding to the proportion of these microorganisms in the SAA group (slanted lines columns). The ratios of Enterobacteriaceae (including *K. pneumoniae, Serratia marcescens, Proteus mirabilis, E. coli, Enterobacter cloacae,* etc.), *H. influenzae, M. catarrhalis* isolated from the SBA group were significantly higher than those in the SAA group (all *P* < 0.05). In contrast, the proportions of *non-fermenting bacteria* (including *Acinetobacter baumannii, Pseudomonas aeruginosa, S. maltophilia, Burkholderia cepacia,* etc.), and *Candida spp.* isolated from the SBA group were significantly lower than those in the SAA group (all *P* < 0.05). There were no statistical differences in the ratios of *S. aureus* and *Streptococcus pneumoniae* between the two groups.

### Blood

**Fig. 3:**
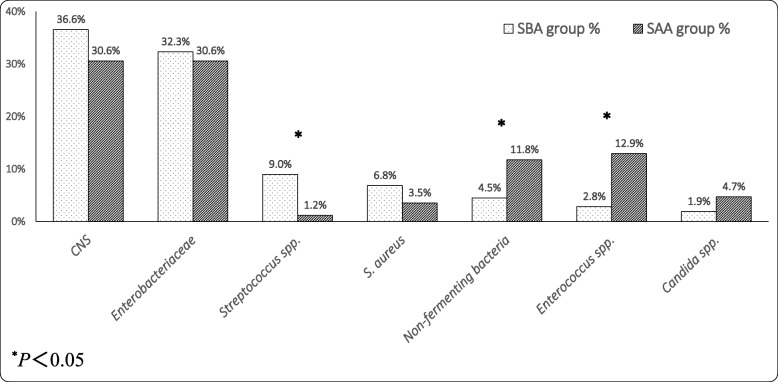
Comparison of microorganisms isolated from blood between SBA (top 7) and SAA groups. Abbreviations: SBA, specimen taken before antibiotic therapy; SAA, specimen taken after antibiotic therapy; CNS, Coagulase-negative staphylococci*; S. aureus, Staphylococcus aureus*

Figure 3 shows the descending order of the top 7 pathogens of the SBA group (dotted columns) corresponding to the proportion of these microorganisms in the SAA group (slanted lines columns). There was a slight difference between the two groups. The ratios of *Streptococcus spp.* (including *Streptococcus viridans, Streptococcus angina,* etc.) from the SBA group were significantly higher than those in the SAA group (*P* < 0.05). The proportions of *non-fermenting bacteria* (including *Acinetobacter baumannii, Pseudomonas aeruginosa,* etc.) and *Enterococcus spp.* (including *Enterococcus faecium*, *Enterococcus faecalis, etc*) isolated from the SBA group were significantly lower than those in the SAA group (all *P* < 0.05). But there was no statistically significant difference between the two groups in the isolation rates of CNS (including *Staphylococcus epidermidis, Staphylococcus humanoidus, staphylococcus capitis, Staphylococcus haemolyticus, staphylococcus warneri,* etc.), Enterobacteriaceae (including *K. pneumoniae, Serratia marcescens*, *Enterobacter cloacae,* etc.)*, S. aureus* and *Candida spp..*

### Urine

**Fig. 4:**
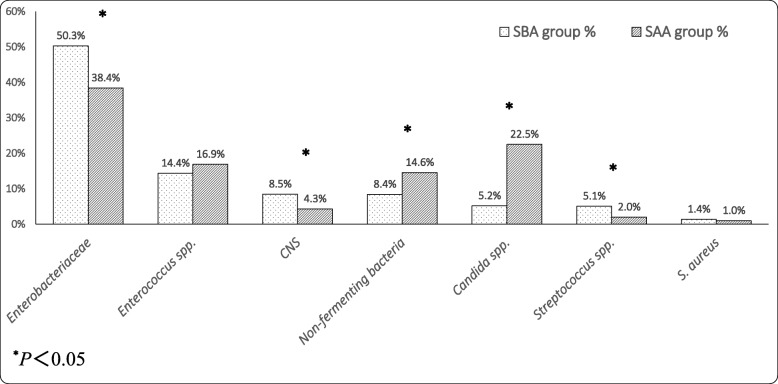
Comparison of microorganisms isolated from urine between SBA (top 7) and SAA groups. Abbreviations: SBA, specimen taken before antibiotic therapy; SAA, specimen taken after antibiotic therapy; CNS, Coagulase-negative staphylococci*; S. aureus, Staphylococcus aureus*

Figure 4 shows the descending order of the top 7 pathogens of the SBA group (dotted columns) corresponding to the proportion of these microorganisms in the SAA group (slanted lines columns). The proportions of *Enterobacteriaceae* (including *E. coli, K. pneumoniae, Proteus mirabilis, Enterobacter cloacae,* etc.), CNS (including *Staphylococcus epidermidis, Staphylococcus haemolyticus,* etc.) and *Streptococcus spp.* (including *Streptococcus agalactiae, Streptococcus viridans,* etc.) isolated from the SBA group were significantly higher than those from the SAA group (all *P* < 0.05). The proportions of *non-fermenting bacteria* (including *Pseudomonas aeruginosa, Acinetobacter baumannii,* etc.) and *Candida spp.* (including *Candida albicans, Candida tropicalis,* etc.) isolated from the SBA group were significantly lower than those of the SAA group (all *P* < 0.05). There was no statistically significant difference found in the remaining microorganisms.

### Body fluid specimens

There were no statistically significant differences in the isolation rates of top 7 major strains between the two groups, including Enterobacteriaceae (including *E. coli, K. pneumoniae, Enterobacter cloacae,* etc.), *Enterococcus spp.*, non-fermenting bacteria(including *Pseudomonas aeruginosa, Acinetobacter baumannii, S. maltophilia,* etc.), CNS (including *Staphylococcus epidermidis, staphylococcus sciuri,* etc.), *Candida spp., S. aureus* and *Streptococcus spp.* between the two groups.

## Discussion

The main finding of this study is that the distribution of microorganisms isolated from specimens taken before and after antibiotic treatment was different, and in most cases, sensitive organisms were more easily isolated before antibiotic therapy, while drug-insensitive organisms such as non-fermenting bacteria and fungi, were more frequently isolated after antibiotic exposure. However, the effect of prior receipt of antibiotics on the pathogen distribution is specimen-dependent and this trend was not obvious in body fluid specimens.

Studies conducted by Montravers et al. [10] and Harbarth et al. [11] showed that starting antibiotic therapy before sample collections may be associated with less-sensitive microorganisms. However, the former only included 76 consecutive patients with ventilator-associated pneumonia [10], and the latter [11] mainly evaluated gram-negative pathogens. Our study expands on these reports, enrolling a large number of patients and analyzing numerous pathogens. To the best of our knowledge, this is the first systematic study evaluating the ecological impact of antimicrobial agents from hospitalized patients based on a large sample size.

From the analysis of the distribution of microorganisms from specimens taken before and after antibiotics therapy, we found that gram-positive organisms had obvious differences between the groups. The results revealed that the percentage of *S. aureus* was higher in the SAA group, while the isolation rates of CNS and *Streptococcus spp.* were higher in the SBA group. The differences in *S. aureus* and *Streptococcus spp.* could be explained by the fact that *Streptococcus spp.* antimicrobial susceptibility is significantly higher than that of *S. aureus*, so the separation rate after treatment may be significantly reduced. However, the reason for the decrease in the separation rate of CNS after the use of antibiotics is unclear. As for gram-negative bacteria, the proportion of non-fermenting bacteria such as *Pseudomonas spp., Acinetobacter spp.*, and *S. maltophilia* isolated from the SAA group was higher than that of the SBA group. It is well known that non-fermenting bacteria are opportunistic nosocomial pathogens and are often found in the hospital environment, frequently with multiple-resistance mechanisms. For example, *Acinetobacter baumannii* has accumulated resistance to most antibiotics [13–15], and in most cases antibiotic treatment should be avoided by virtue of low potential virulence [16]. Additionally, this study also found that the separation rates of *H. influenzae* and *M. catarrhalis* were significantly lower after antibiotics administration. It has been speculated that the two bacteria are pathogens of typical community sources, and that they are generally sensitive to most antibacterial agents as is consistent with the present results [17], although recent studies have shown that resistance in these two bacteria is increasing [18]. In fact, the differences of microorganism distribution between the groups could partially be explained by the antibiotics prescribed before sampling in SAA group. It was found that cephalosporins, β-lactamase inhibitor complex and carbapenems accounted for 82.3% of all drugs used in SAA group, which may exert effect on the final microbial results. For example, in view of the high degree of drug resistance of the non-fermenting bacteria, antibiotics may kill relatively more sensitive pathogens, such as *streptococci spp.*, *H. influenzae*, and *M. catarrhalis*. Besides, our results suggested that insensitive bacteria and common multidrug-resistant (MDR) bacilli in hospitals are more likely to be isolated after antibiotic exposure, which may further result in the use of unnecessary antibiotics and provide selective pressure in favor of MDR organisms.

On the other hand, further subgroup analysis based on specimen types also showed significant differences of microbes derived from respiratory secretions, blood or urine samples taken before or after antibiotic exposure. For example, the isolates of non-fermenting bacteria and *enterococcus spp.* in blood samples were different between the two groups, with separation rates increasing after antibiotic therapy. The same phenomenon occurs in urine cultures. The subgroup analysis of urine samples showed that differences between the two groups were significant. Bidell et al. [19] reported that *Pseudomonas aeruginosa* isolated from the urine of patients suffering from urinary tract infections is increased in patients with prior antibiotic exposures, but no differences could be found in other pathogens. However, we found that the rates of common microbes such as *Enterobacteriaceae,* CNS*,* and *Streptococcus spp.* decreased after exposure to antimicrobial agents, whereas the isolation rates of non-fermenting bacteria and *Candida spp.* in the SAA group were higher than those isolated from the SBA group. As far as we know, the latter two are not sensitive or affected by commonly used antimicrobial agents. As for respiratory secretions, we found that the proportions of common community acquired pneumonia pathogens isolated from respiratory secretions in both groups were less than those reported before [20, 21]. Antibiotics having been prescribed before transferring to our hospital, a regional medical center, may account for this difference. Prior antibiotic treatment may reduce respiratory pathogen yield and increase false-positive results [9, 22–26]. In fact, frequent and inappropriate use of antibiotics in primary health care settings in China is a serious problem [27].

However, prior antibiotic exposure seemed to have no significant effect on microorganisms isolated from body fluid specimens. Indeed, the few studies investigating the yield of these clinical specimens after antibiotic exposure are inconsistent. In a study with 2740 episodes of orthopedic infections, Al-Mayahi et al. [28] showed prior antibiotic use, even a single-dose prophylactic administration, was associated with subsequent culture-negative results, non-fermenting rods and resistant skin commensals. Another study conducted by Shahi et al. [7] also demonstrated the negative effect of antibiotic exposure on the culture results. However, some reports suggested that prescribing antibiotic treatment before operative sampling dose not increase the risk of culture-negative results [29, 30]. Therefore, the role of antibiotic administration in these results is unknown, and further research to help identify possible correlations is needed.

Besides, the study also found the isolation rate of fungi, especially *Candida spp.*, was significantly higher after antibiotic exposure. Usually, *Candida spp.* can be isolated from airway samples but are not considered to play a significant role in acute illness [31]. Other authors have found that fungal airway colonization is frequent in patients with mechanical ventilation [31–34]. Therefore, the clinical significance of the higher isolation rate of fungi after antibiotic exposure needs further evaluation.

There were several limitations to this study. First, this was a retrospective study, thus subjecting our results to multiple biases, such as selection bias. Second, despite a large sample, the study was limited only to a single center, which means that the results may not be applicable to other settings. Third, this study was not able to analyze the use of antimicrobial agents before admission and the perioperative antibiotics, which may result in some patients in unsuitable grouping. Besides, we did not analyze whether the antimicrobial agents used for treatment were effective against the cultured microorganisms, and therefore could not speculate on their causal relationship. Finally, this study also did not identify whether these microorganisms were causative pathogens.

## Conclusion

we showed that clinical specimens that are easier to collect, such as blood and urine, were more likely to be collected before antibiotic therapy. Further, in most cases, sensitive organisms were more easily isolated before antibiotic use, while drug-insensitive organisms, such as non-fermentative bacteria and fungi, were more frequently isolated after antibiotic exposure. However, this trend was not obvious in body fluid specimens. Further clinical studies are needed to analyze the effect of antimicrobial agents on changes in antimicrobial susceptibility.

## Data Availability

All data generated or analysed during this study are included in this published article.

## Abbreviations

aCDSS: Computerized antimicrobial decision-support system
ANOVA: Analysis of variance
*CNS*: Coagulase-negative Staphylococcus
*E. coli*: *Escherichia coli*
EMRS: Electronic medical record system
*H. influenza*: *Haemophilus influenzae*
ICU: Intensive care unit
IQR: Interquartile range
*K. pneumonia*: *Klebsiella pneumoniae*
*M. catarrhalis,*: *Moraxella catarrhalis*
*S. aureus*: *Staphylococcus aureus*
*S. maltophilia*: *Stenotrophomonas maltophilia*
SAA: Specimen taken after antibiotic therapy
SBA: Specimen taken before antibiotic therapy
SD: Standard deviation
